# Corrosion Behaviour of S32101 (1.4162—X2CrMnNiN21-5-1) Stainless Steel in Pulping Liquors

**DOI:** 10.3390/ma18091921

**Published:** 2025-04-24

**Authors:** Banele Siyabonga Kheswa, David Whitefield, Herman Potgieter, Michael Bodunrin

**Affiliations:** Materials Utilisation Group (MUG), School of Chemical and Metallurgical Engineering, University of the Witwatersrand, Private Bag X3, Wits, Johannesburg 2050, South Africa; david.whitefield@wits.ac.za (D.W.); michael.bodunrin@wits.ac.za (M.B.)

**Keywords:** corrosion, cyclic polarisation, pulp liquors, acids, salts

## Abstract

The corrosion behaviour of lean duplex S32101 (1.4162—X2CrMnNiN21-5-1) stainless steel was assessed in various corrosive environments relevant to the pulp and paper industry. Electrochemical techniques, including open-circuit potential measurements and cyclic polarisation, were used to evaluate the corrosion resistance of S32101 stainless steel in various acidic, saline, and industrial liquors such as black, green, and white liquors, as well as dissolved chlorine dioxide bleaching solutions. To evaluate the extent of damage and corrosion mechanisms, post-exposure surface analysis was conducted using scanning electron microscopy (SEM). The results showed that S32101 experienced pitting corrosion in chloride-containing solutions, particularly in salt and acidified-salt environments. Corrosion rates increased with rising temperatures across all solutions. The highest corrosion rate of 3.17 mm/yr was observed in the highly alkaline white liquor at 50 °C, whilst chlorine dioxide induced the least aggressive effects at all temperatures. The suitability of S32101 stainless steel in handling pulp and paper liquors is shown in its corrosion resistance against the bleaching medium and low-temperature saline solutions, but it is not recommended for prolonged exposure to high alkaline liquors or chloride-rich solutions.

## 1. Introduction

Corrosion is a significant global challenge, affecting infrastructure, industrial equipment, and structural components across various sectors. The annual cost of corrosion is estimated to be approximately 3–4% of the global GDP, amounting to trillions of dollars [1]. These costs arise from material degradation, maintenance, premature equipment failure, and associated safety risks. In addition to the economic burden, corrosion poses serious environmental and safety concerns. Structural failures due to corrosion can lead to hazardous leaks, environmental contamination, and catastrophic accidents, especially in industries handling chemicals, energy production, and water treatment. While corrosion is inevitable, effective material selection, protective coatings, and corrosion-resistant alloys can significantly mitigate its impact.

The pulp and paper industry presents one of the most challenging environments for material durability, particularly regarding corrosion resistance. Various pulp and paper production stages, including pulping, chemical recovery, and bleaching, involve exposure to highly corrosive substances such as sulphur compounds, chlorine-based chemicals, and alkaline solutions [2,3]. In alkaline pulping mills, the cooking liquor consists mainly of sodium hydroxide and sodium sulphate, the combination of which is an active alkali [4]. The digesters operate at temperatures and pressures of about 180 °C and 890 kPa. Unlike in sulphite pulping mills, where the corrosion is localised, and temperature-dependent, the corrosion in alkaline pulp mills is primarily general. The digesters containing highly caustic solutions at their associated temperatures, have been found to suffer from general and stress corrosion cracking [5]. A study on the contrast in the effects of acidity and alkalinity on corrosion behaviour showed that general and pitting corrosion occurred in acidic environments. In contrast, in alkaline environments, general corrosion was more prevalent [6]. Some manufacturers have reported difficulties in their causticising systems and their white liquors. Amidst the difference in problems that different alkaline pulp mills face, the survey performed by Whitney [7] indicated that the use of nickel-alloyed and high chromium-content steels minimised the corrosion damage. Therefore, selecting corrosion-resistant materials is critical to ensuring the longevity and efficiency of industrial components, including digesters, pipelines, and processing vessels.

Among the different stainless steels utilised in the pulp and paper industry, lean duplex stainless steels have gained prominence due to their cost-effectiveness and enhanced corrosion resistance in aggressive environments. UNS S32101, a second-generation lean duplex stainless steel, has emerged as a viable alternative to conventional austenitic grades such as 304L and 316L, particularly in environments where chloride-induced corrosion and caustic embrittlement are prevalent [8,9]. S32101 contains lower nickel and molybdenum content than standard duplex and super duplex stainless steels, making it a more economical choice while maintaining adequate corrosion resistance through the addition of nitrogen [10]. The effect of white liquor on the corrosion resistance of duplex stainless steel has been extensively studied. White liquor is the most alkaline of the pulping liquors and contains the highest concentrations of caustic and sulphide-containing species and is therefore considered to be the most aggressive of all pulping media [11]. Bhattacharya and Singh [11] studied the effects of high pH caustic and alkaline sulphide at a range of temperatures on the corrosion properties and electrochemical behaviour for different duplex stainless steels (UNS S32304, UNS S32101, and UNS S32205). Making comparisons of pure iron, chromium, nickel, and molybdenum with standard duplex stainless steel (UNS S32205) by analysing these metals’ polarisation behaviours in different environments, a study on the role of alloying elements in steels was deduced. The corrosion rates of the duplex stainless steels increased with the addition of sulphur. The presence of sulphur in the passive layer caused the formation of metal sulphur compounds, which induced a less protective film than an oxide film. As a result, the standard duplex stainless steels experienced higher corrosion rates (mostly general corrosion) than the lean duplex grades, with the latter having high corrosion resistance in a caustic sulphide environment. The high corrosion resistance of the lean duplex grades can be attributed to the forming of a more stable magnetite and awaruite passive film, which resulted in low corrosion rates [11,12].

In the Southern African context, the challenges associated with corrosion in pulp and paper mills are exacerbated by the region’s reliance on ageing infrastructure and the need for cost-effective solutions. A case study from a South African pulp and paper mill revealed significant corrosion concerns in continuous and batch digesters, bleaching facilities, and pipelines handling white, black, and green liquors. Fibreglass materials have been adopted in some applications, but their structural limitations under mechanical stress necessitate the exploration of more robust metallic alternatives.

This study investigates the corrosion behaviour of S32101 stainless steel in various pulp and paper environments, focusing on its performance in key processing stages where exposure to corrosive liquors is most severe. By understanding the corrosion behaviour affecting this material, this research aims to provide insights into its suitability for long-term industrial application, thereby contributing to more reliable material selection strategies in Southern African pulp and paper facilities. The findings will help address unplanned downtimes due to corrosion-related failures and promote adopting cost-effective and durable stainless steel solutions in the industry.

## 2. Materials and Methods

### 2.1. Materials

The lean duplex stainless steel grade S32101 utilised in this study is known for its high corrosion resistance and was procured in a cold-rolled condition with a thickness of 6 mm. The composition of the S32101 stainless steel grade is shown in Table 1. The alloy was machined into 8 mm × 8 mm samples for corrosion testing. The samples were attached to a wire using aluminium tape and then embedded in an insulating epoxy resin. Typical curing times (in air) for the samples in the resin were between two to three hours. The surfaces of the samples were ground to 4000 grit in the following intervals: 500, 800, 1200, 2000, and 4000, and then cleaned using water and alcohol before being dried.

The electrolyte solutions prepared in the laboratory included 0.1 M HCl, 1 M H_2_SO_4_, and 3.5% NaCl for corrosion testing. An acid chloride mixture was also formulated by combining 0.2 M HCl and 7% NaCl solutions to obtain a 0.1 M HCl-acidified 3.5% NaCl solution. Process liquor samples obtained from a South African pulp and paper mill included black liquor, white liquor, green liquor, and dissolved ClO_2_ as the bleaching medium. The compositions of these solutions are presented in Table 2.

### 2.2. Electrochemical Testing

The electrochemical tests were conducted using a three-electrode cell setup (Metrohm, Johannesburg, South Africa) connected to a potentiostat for open-circuit potential (OCP) and cyclic polarisation measurements. The setup included a silver/silver chloride (Ag/AgCl) electrode as the reference electrode (RE), a platinum rod as the counter electrode (CE), and the test sample as the working electrode (WE). A water bath was used to maintain elevated test temperatures of 30 °C, 40 °C, and 50 °C, with temperature stability controlled within ±1 °C.

OCP and cyclic polarisation curves were obtained using NOVA electrochemical software (version 2.1.6, Metrohm, Johannesburg, South Africa) integrated with a PGSTAT20 Autolab Potentiostat. The samples were conditioned at OCP before recording the cyclic polarisation scans for a period of typically 2 h. Cyclic polarisation tests were performed using a start potential of −0.5 V vs. the reference electrode, a stop potential of 1.7 V vs. the reference electrode and a scan rate of 0.167 mV/s. Corrosion rate analysis was conducted using the NOVA software (version 2.1.6, Metrohm, Johannesburg, South Africa), which facilitated the generation of Tafel plots. Linear regions of the anodic and cathodic branches were selected for regression fitting, with the intersection of the regression lines used to determine the corrosion potential and corrosion current.

### 2.3. Scanning Electron Microscopy

The corroded samples were examined using a Carl Zeiss Sigma field emission scanning electron microscope (FE-SEM) model-SIGMA-03-39 (Zeiss Microscopy, Jena, Germany) operated in both back-scattered electron (BSE) and secondary electron (SE) imaging modes. Energy-dispersive X-ray spectroscopy (EDS) to determine the average composition was conducted on the corroded surfaces to analyse the composition of the corrosion products. The potential value was set at 20 kV, and the current was 2.4 µA.

## 3. Results

This section presents the open circuit potential (OCP) versus time curves, the cyclic polarisation curves, and the scanning electron images of duplex stainless steel samples immersed in different corrosion media at various temperatures.

### 3.1. Open Circuit Potential

**Figure 1:**
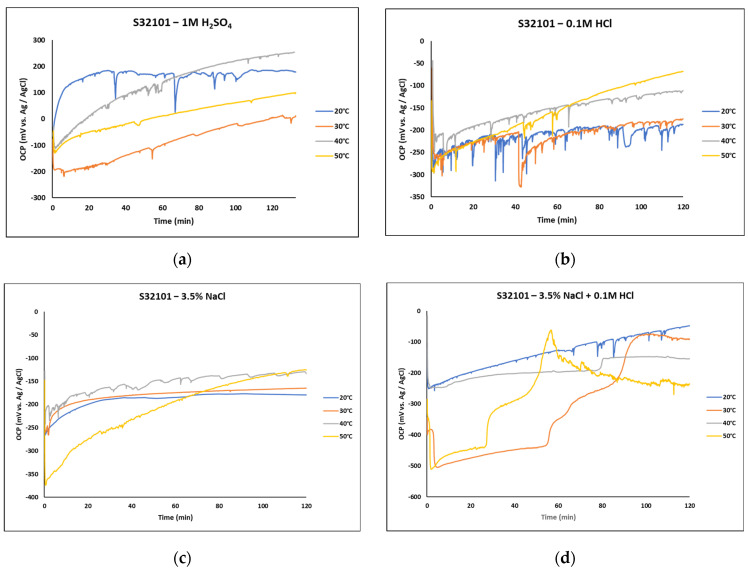
Progression of OCP for S32101 stainless steel immersed for 120 min in different laboratory solutions at various temperatures. (**a**) 1 M H_2_SO_4_; (**b**) 0.1 M HCl; (**c**) 3.5 wt% NaCl; and (**d**) 3.5 wt% NaCl + 0.1 M HCl.

Figure 1 presents the OCP curves of S32101 stainless steel in both laboratory-prepared strong reducing acids and salt solutions. Overall, the alloy exhibited an increasing OCP trend toward anodic potentials with prolonged immersion, regardless of temperature. This upward trend can be attributed to the spontaneous formation and growth of a chromium oxide passive film on the surface of the alloy upon exposure to the corrosive media [14].

Despite this general trend, the influence of the corrosive environment and temperature on passive film stability is evident under specific conditions. In Figure 1b, OCP fluctuations are more pronounced in 0.1 M HCl at all temperatures, indicating a less stable passive film compared to other solutions—1 M H_2_SO_4_ (Figure 1a), 3.5 wt% NaCl (Figure 1c), and 3.5 wt% NaCl + 0.1 M HCl (Figure 1d). The destabilising effect of HCl is further highlighted when comparing the salt solutions (Figure 1c,d). In 3.5 wt% NaCl + 0.1 M HCl, the OCP shifted towards cathodic potentials after 60 min of immersion at 50 °C, whereas in 3.5 wt% NaCl, OCP continued to increase at the same temperature.

All chloride-containing solutions (Figure 1c,d) exhibited lower OCP values than the sulphate-containing solution (Figure 1a). However, a stable passive film was also observed in 3.5 wt% NaCl at 20 °C and 30 °C.

The effect of temperature on OCP behaviour was inconsistent across the solutions. In 1 M H_2_SO_4_, the highest OCP value was recorded at 20 °C throughout the immersion period. Similarly, in 3.5 wt% NaCl + 0.1 M HCl, OCP was highest at 20 °C. Conversely, in 0.1 M HCl and 3.5 wt% NaCl, the highest OCP values were observed at 40 °C and 50 °C.

**Figure 2:**
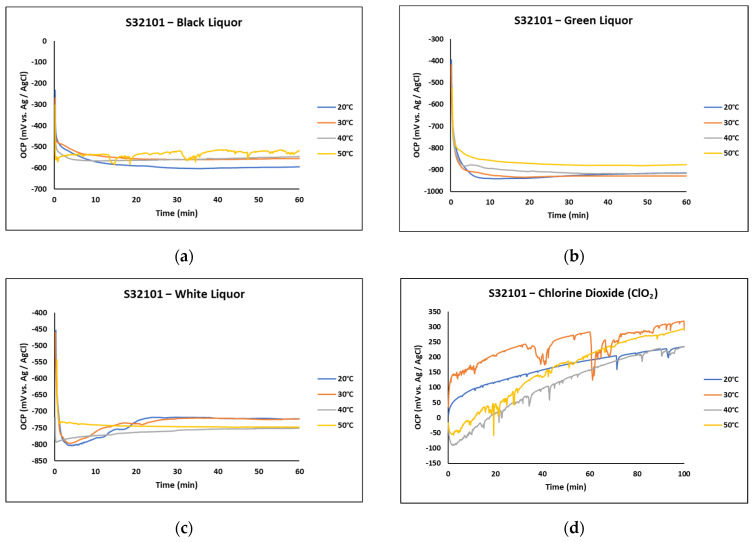
Progression of OCP for S32101 stainless steel immersed for 120 min in different pulp and paper solutions at different temperatures. (**a**) Black liquor; (**b**) green liquor; (**c**) white liquor; and (**d**) bleaching solution.

Figure 2 presents the OCP curves of S32101 stainless steel immersed in various pulp and paper liquors at different temperatures. Unlike the trends observed in the laboratory-prepared acidic and salt solutions (Figure 1), the OCP in black, green, and white liquors (Figure 2a–c) exhibited an opposite behaviour. The OCP values declined rapidly and stabilised at significantly lower cathodic potentials: below −500 mV vs. Ag/AgCl in black liquor, −800 mV vs. Ag/AgCl in green liquor, and −700 mV vs. Ag/AgCl in white liquor. This sharp decrease suggests that the passive film on the alloy surface dissolved rapidly in these solutions, increasing its susceptibility to corrosion.

The only exception among the pulp and paper liquors was chlorine dioxide, which followed a trend similar to that observed in Figure 1. In this medium (Figure 2d), the OCP of S32101 stainless steel increased with exposure time. This behaviour may be attributed to the strong oxidising nature of chlorine dioxide, which likely promoted the formation and growth of a chromium oxide passive film on the alloy surface. However, fluctuations in the OCP curves indicate cycles of film dissolution and repassivation, resembling the trends seen in Figure 1.

Temperature had a marginal effect on the OCP of S32101 in black, green, and white liquors, as the recorded values remained within a similar range for each liquor. In chlorine dioxide, however, the highest OCP values were observed at 30 °C and 50 °C.

### 3.2. Cyclic Polarisation

**Figure 3:**
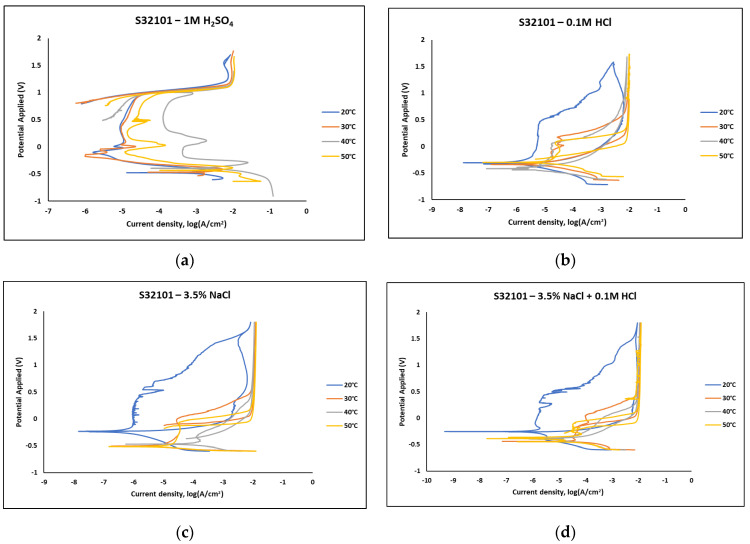
Polarisation curves of S32101 stainless steel exposed to (**a**) 1 M H_2_SO_4_; (**b**) 0.1 M HCl; (**c**) 3.5 wt% NaCl; and (**d**) 3.5 wt% NaCl + 0.1 M HCl at different temperatures.

Figure 3 presents the polarisation curves obtained from cyclic polarisation scans of S32101 stainless steel immersed in laboratory-prepared acidic and salt solutions. The corrosion performance of the alloy is strongly influenced by the type of corrosive medium. In the sulphate-containing solution (1 M H_2_SO_4_, Figure 3a), a distinct active–passive transition is observed at all temperatures. However, this behaviour is absent in the chloride-containing solutions—0.1 M HCl, 3.5 wt% NaCl, and 3.5 wt% NaCl + 0.1 M HCl (Figure 3b–d), where only spontaneous passivation occurs with increasing polarisation, and the active “nose” is absent. Additionally, the sulphate-containing medium exhibits a negligible hysteresis loop, with the reverse scan aligning directly with the forward scan, indicating no clear repassivation potential and, passively, the dominance of uniform corrosion [14,15]. In contrast, the chloride-containing solutions display large hysteresis loops, with repassivation potentials close to the corrosion potential, especially at 20 °C. This suggests a higher susceptibility to pitting corrosion in chloride environments compared to the sulphate solution. Across all media, transient currents appear in the passive region, indicating that the passive film is compromised by ionic attack. These transient currents are consistent with the fluctuating OCP trends observed in Figure 1.

The passivation current is generally lower at 20 °C than higher temperatures, particularly in chloride-containing solutions. To further elucidate the effect of corrosive media and temperature on the electrochemical behaviour of S32101 stainless steel, key corrosion parameters were extracted from the polarisation curves (Figure 3) and are presented in Table 3, Table 4, Table 5 and Table 6. The corrosion potential of the alloy in acidic media (1 M H_2_SO_4_ and 0.1 M HCl) remains similar, at approximately −450 mV across all test temperatures. However, in salt solutions (3.5 wt% NaCl and 3.5 wt% NaCl + 0.1 M HCl), the corrosion potential is slightly higher at 20 °C compared to 30–50 °C. These trends suggest that the corrosion tendency of S32101 stainless steel in acidic solutions remains consistent across temperatures, while in salt solutions, susceptibility to corrosion increases at temperatures above 20 °C. Furthermore, both the critical current density and corrosion rate increase significantly with rising temperatures in all media, indicating that elevated temperatures accelerate corrosion processes.

**Figure 4:**
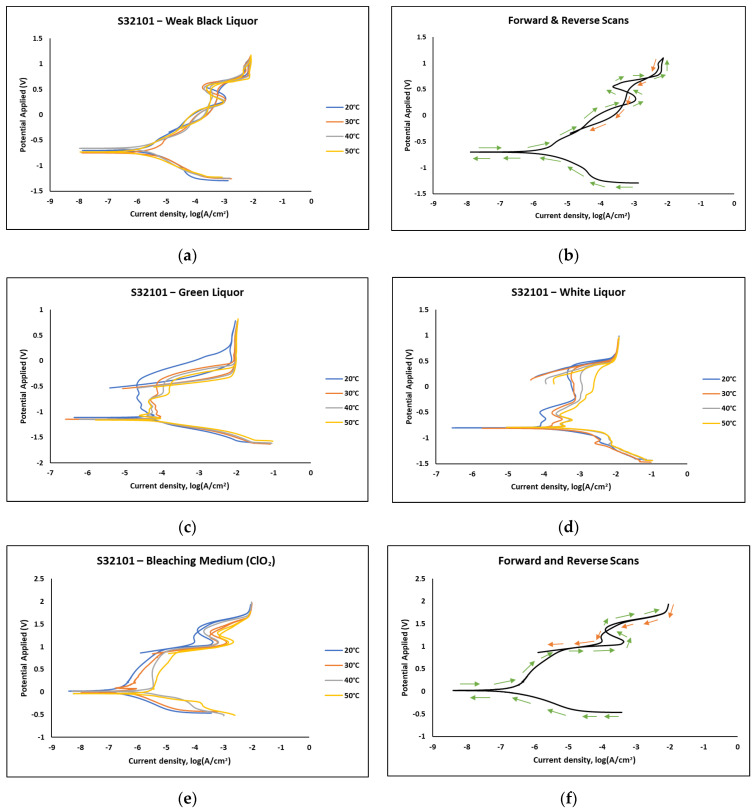
Polarisation curves of S32101 stainless steel exposed to (**a**) black liquor; (**b**) black liquor with directions; (**c**) green liquor; and (**d**) white liquor (**e**) chlorine dioxide; and (**f**) chlorine dioxide with directions at different temperatures.

Figure 4 presents the polarisation curves obtained from cyclic polarisation experiments in various pulp and paper liquors. No passivation was observed in black liquor (Figure 4a), as indicated by the nearly identical forward and reverse scans (Figure 4b), suggesting uniform corrosion as the dominant mechanism. In contrast, the alloy exhibited a small passivation region in green, white, and chlorine dioxide environments, with only minor differences in passivation current and potential across different temperatures. However, a slightly superior performance was noted at 20 °C in these three media. Unlike the chloride-containing solutions in Figure 3, which displayed large hysteresis loops indicative of pitting corrosion, the pulp and paper liquors, including the chloride-containing bleaching solution, did not show such behaviour.

While the polarisation scans indicate minimal differences in alloy behaviour across the pulp and paper solutions, further insights can be drawn from the corrosion parameters derived from Figure 4, presented in Table 7, Table 8, Table 9 and Table 10 for black, green, and white liquors, and chlorine dioxide solution, respectively. In each medium, corrosion potential remained largely insensitive to temperature changes, whereas critical current density and corrosion rate increased with rising temperature. This aligns with the OCP trends in Figure 4, where the alloy exhibited similar behaviour regardless of temperature variations.

Among the pulp and paper liquors, S32101 stainless steel showed the highest tendency to corrode in green liquor, as indicated by its more negative corrosion potential. Conversely, the higher corrosion potential observed in chlorine dioxide suggests that the alloy is least susceptible to corrosion in this medium. Additionally, chlorine dioxide exhibited the lowest corrosion rate, decreasing by at least a factor of 10 compared to the other liquors. This further corroborates the OCP results in Figure 4, where passive film growth was observed in chlorine dioxide, in contrast to the dissolution seen in the other pulp and paper liquors. The oxidising nature of chlorine dioxide likely contributes to this improved corrosion resistance.

### 3.3. SEM Images of Corroded Surfaces

**Figure 5:**
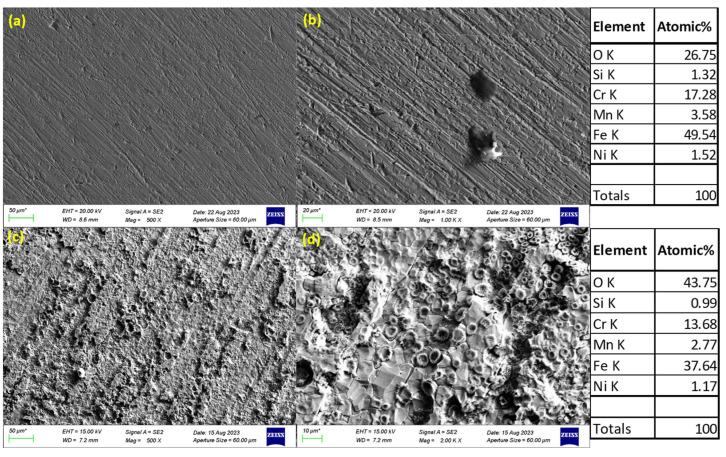
SEM images of S32101 in 1 M H_2_SO_4_ at 20 °C (**a**,**b**) and 50 °C (**c**,**d**).

**Figure 6:**
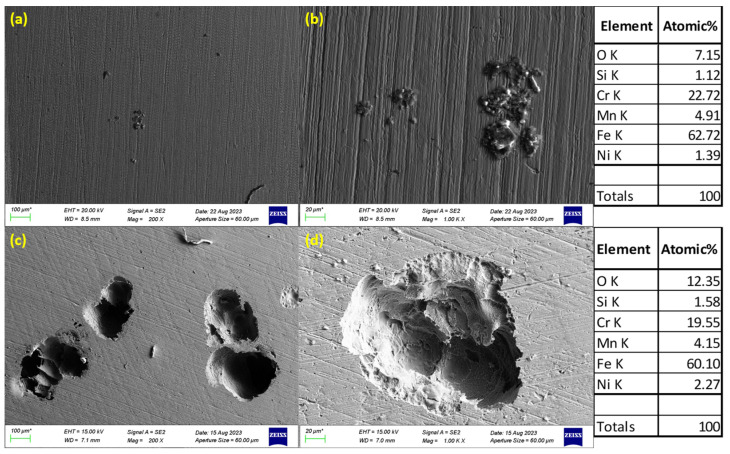
SEM images of S32101 in 0.1 M HCl at 20 °C (**a**,**b**) and 50 °C (**c**,**d**).

**Figure 7:**
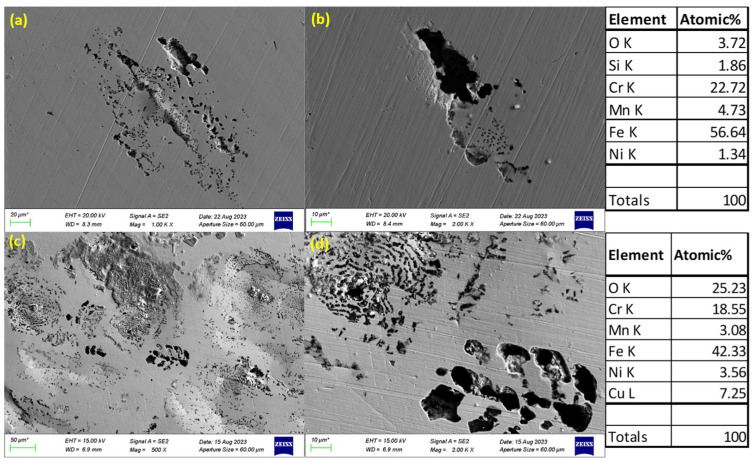
SEM images of S32101 in 3.5% NaCl at 20 °C (**a**,**b**) and 50 °C (**c**,**d**).

**Figure 8:**
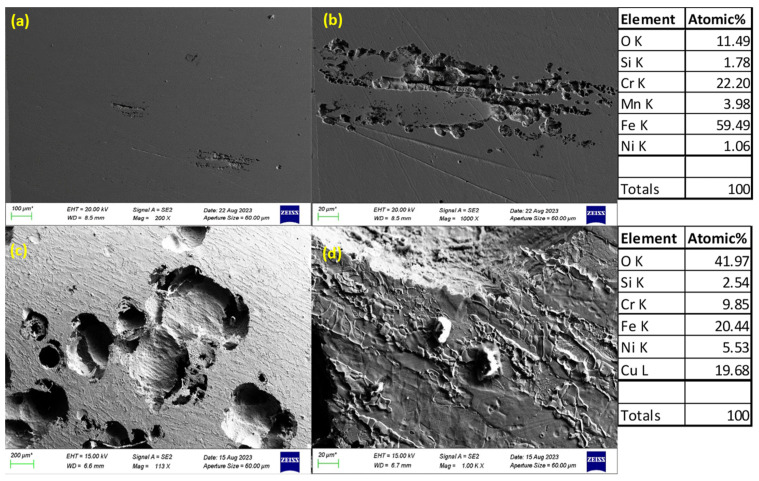
SEM images of S32101 in 3.5% NaCl + 0.1 M HCl at 20 °C (**a**,**b**) and 50 °C (**c**,**d**).

The surface morphology of S32101 stainless steel was analysed using SEM in secondary electron imaging or back-scattered mode after corrosion experiments in various solutions. Selected SEM images at 20 °C and 50 °C are presented in Figure 5, Figure 6, Figure 7 and Figure 8 to confirm the type or nature of corrosion in each environment. These images show the corroded surfaces of the alloy after exposure to 1 M H_2_SO_4_, 0.1 M HCl, 3.5 wt% NaCl, and 3.5 wt% NaCl + 0.1 M H_2_SO_4_ solutions.

In the sulphuric acid solution (Figure 5), extensive corrosion product formation indicates that uniform corrosion was the predominant mechanism. In contrast, all chloride-containing solutions (Figure 6, Figure 7 and Figure 8) exhibited pits of varying sizes, confirming the occurrence of pitting corrosion. These findings align with the cyclic polarisation results in Section 3.2, where hysteresis loops were observed only in the reverse scan of polarisation curves of the chloride-containing solutions, indicating susceptibility to pitting.

Energy-dispersive X-ray spectroscopy (EDS) analysis revealed that oxygen, iron, and chromium were consistently present in significant amounts, suggesting that the primary corrosion products on the alloy surface are iron and chromium oxides.

The influence of temperature on corrosion behaviour is evident in the SEM images. At 20 °C, the volume of corrosion products and the number of pits are noticeably lower compared to those observed at 50 °C. This confirms the temperature dependence of the corrosion rate, as reflected in the critical current density and corrosion rate data presented in Table 3, Table 4, Table 5 and Table 6, where corrosion rates generally increased with rising temperatures.

**Figure 9:**
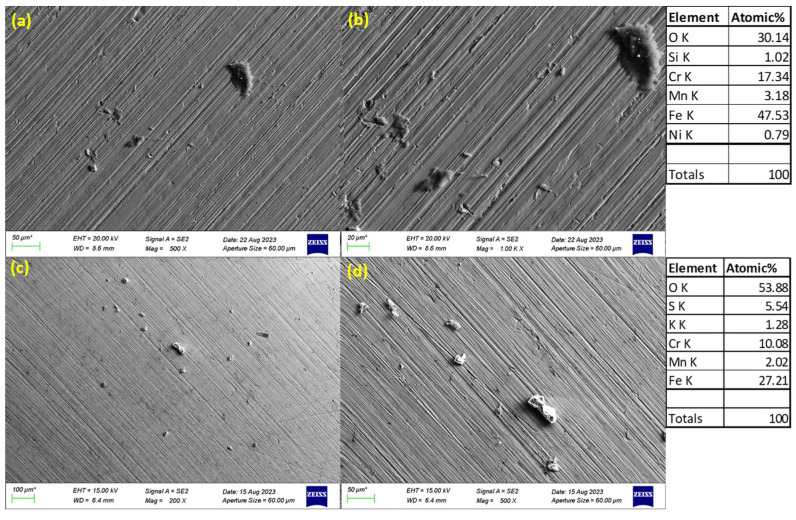
SEM images of S32101 in weak black liquor at 20 °C (**a**,**b**) and 50 °C (**c**,**d**).

**Figure 10:**
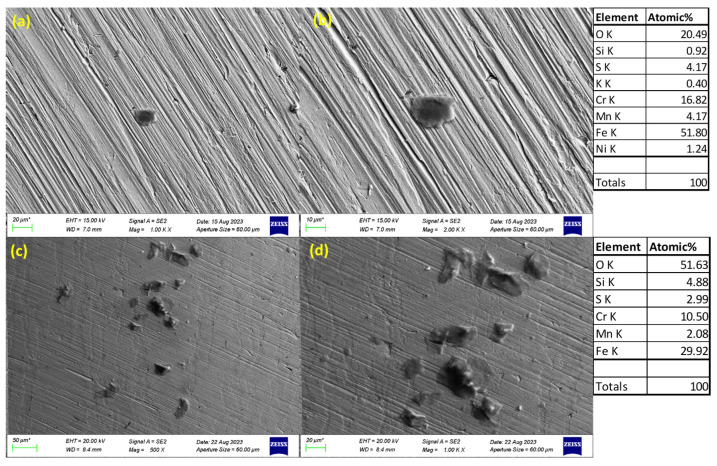
Scanning electron microscopy images of S32101 in green liquor at 20 °C (**a**,**b**) and 50 °C (**c**,**d**).

**Figure 11:**
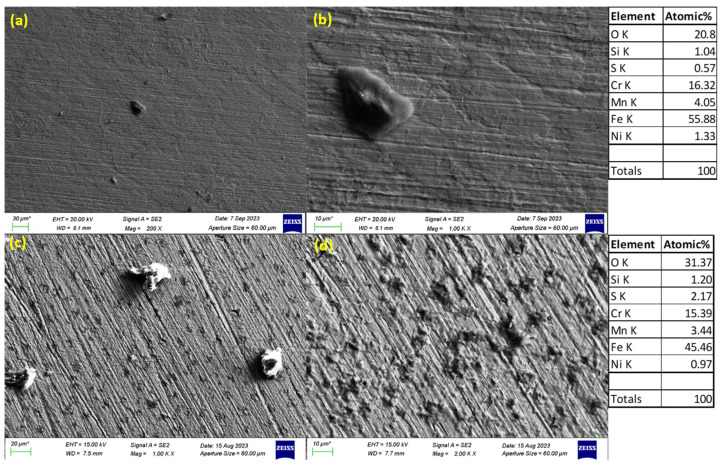
Scanning electron microscopy images of S32101 in white liquor at 20 °C (**a**,**b**) and 50 °C (**c**,**d**).

**Figure 12:**
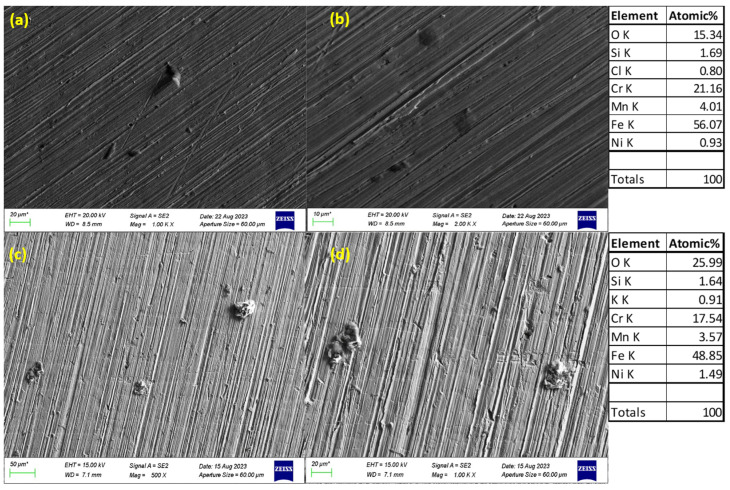
Scanning electron microscopy images of S32101 in bleaching medium (ClO_2_) (**a**,**b**) and 50 °C (**c**,**d**).

Figure 9, Figure 10, Figure 11 and Figure 12 present SEM images of S32101 stainless steel after exposure to pulp and paper liquors—black, green, white, and chlorine dioxide solutions. The images reveal only corrosion products on the alloy surface, with no visible pits indicative of pitting corrosion. This confirms that uniform corrosion is the predominant degradation mechanism in these environments. These findings align with the cyclic polarisation results in Figure 3, where the absence of large hysteresis loops in the reverse scan indicated low susceptibility to localised attack.

S32101 stainless steel experienced less severe corrosion in the pulp and paper liquors than the laboratory-prepared acidic and salt solutions. Similarly to the laboratory-prepared media, the corrosion products are essentially iron and chromium oxides. The corrosion products appeared thinner and less extensive, supporting the lower corrosion rates reported in Table 7, Table 8, Table 9 and Table 10, as opposed to the higher rates observed in the acidic and salt solutions (Table 3, Table 4 and Table 6).

The SEM images also validate the influence of temperature on corrosion rates in pulp and paper liquors. At higher temperatures, increased corrosion led to greater deposition of corrosion products on the alloy surface. This trend is particularly evident in white liquor, which exhibited the highest corrosion rate among the tested liquors. In contrast, the least corrosion products were observed on samples exposed to chlorine dioxide (Figure 12), consistent with its significantly lower corrosion rate—at least a factor of 10 lower, as indicated in Table 10.

## 4. Discussion

The corrosion performance of S32101 stainless steel in various solutions encountered in the pulp and paper industry can be assessed based on its response to the dominant ions present in solutions and the effect of different operating temperatures. The primary corrosion resistance mechanism of S32101 stainless steel is the formation of a protective chromium oxide film on its surface when exposed to corrosive media [14,16]. However, once the integrity of this passive film is compromised, the alloy becomes susceptible to both uniform and localised corrosion.

The experimental results indicate that S32101 stainless steel exhibits different corrosion behaviours depending on the corrosive medium. Pitting corrosion was predominant in chloride-containing solutions, except in chlorine dioxide, which is an oxidising agent [17]. As an oxidising agent, chlorine dioxide favours more passive film growth than the strong acids, as witnessed by its tendency toward the highest OCP and corrosion potentials amongst the test solutions (Figure 2 and Table 10, respectively). In hydrochloric acid, numerous pits of varying sizes formed on the corroded alloy surface (Figure 6, Figure 7 and Figure 8). However, in strong reducing acid containing sulphate ions, pitting corrosion was not observed; instead, uniform corrosion was the dominant mechanism (Figure 5). These findings align with previous studies, which have shown that stainless steels are more prone to pitting corrosion in chloride environments than in sulphate-containing solutions [18,19,20,21]. The cyclic polarisation scans (Figure 3b–d) revealed transient currents and large hysteresis loops in chloride-containing solutions, indicating susceptibility to pitting corrosion, which was further confirmed by SEM images (Figure 6, Figure 7 and Figure 8).

An exception to this trend was observed in the bleaching solution, which primarily consists of chlorine dioxide. Stainless steels have been reported to exhibit high resistance to pitting in bleach plant chlorine dioxide due to its oxidising nature, which promotes thickening of the passive film rather than its dissolution [22]. This effect is particularly significant when the pH of the chlorine dioxide solution is near neutral. However, at lower or higher pH values, stainless steel may still be susceptible to pitting [23,24]. Other solutions tested in this study, including black, green, and white liquors, showed predominantly uniform corrosion, with no evidence of pitting on the alloy surface after exposure (Figure 9, Figure 10, Figure 11 and Figure 12). Additionally, the cyclic polarisation scans in these solutions exhibited no hysteresis loops, further confirming the absence of localised attack.

Regarding corrosion rates, the laboratory-prepared solutions were more aggressive than black, green, and white liquors due to the presence of chloride and sulphate ions in the former. Furthermore, the presence of organic compounds such as lignin in black and green liquors may have contributed to corrosion inhibition [25,26,27]. The highest corrosion rates were observed in white liquor, which contains at least 90% NaOH. When the NaOH concentration exceeds 1 M, it can dissolve the passive chromium oxide film on the stainless steel surface, significantly accelerating corrosion [28,29]. This sensitivity to solution composition was evident in the results, where white liquor, containing highly corrosive NaOH, led to a much higher corrosion rate than black and green liquors, which primarily consist of Na_2_CO_3_, a less aggressive alkaline solution [30].

The influence of temperature on the corrosion susceptibility of S32101 stainless steel was relatively minimal, as indicated by the similar corrosion potentials observed across different temperatures in each solution (Table 3, Table 4, Table 5, Table 6, Table 7, Table 8, Table 9 and Table 10). Additionally, the open-circuit potential trends (Figure 1 and Figure 2) remained largely consistent across temperatures, with only slight variations. However, temperature significantly impacted corrosion rates, with a general trend of increasing corrosion rate with rising temperature (Table 3, Table 4, Table 5, Table 6, Table 7, Table 8, Table 9 and Table 10). This trend is further validated by SEM images (Figure 5 and Figure 11), which show a greater extent of corrosion damage at 50 °C compared to 20 °C. Previous studies on other stainless steel grades have demonstrated that increasing temperature accelerates electrochemical reactions, leading to a more defective passive film and higher corrosion rates [31,32,33]. The instability of chromium at elevated temperatures results in a thinner and less protective oxide layer, making the alloy more vulnerable to corrosion [34].

Since the maximum permissible corrosion rate in the pulp and paper industry is 5 mpy (0.127 mm/yr) [35], it is crucial to determine suitable environments for the application of S32101 stainless steel, especially considering its potential as a cost-effective alternative to traditional 304L and 316L stainless steel [36]. The findings suggest that S32101 stainless steel may be suitable for use in saline environments, provided the salt solution is not acidified, and operating temperatures do not exceed 40 °C. The corrosion rate of S32101 in 3.5 wt% NaCl at temperatures below 40 °C was at least ten times lower than the industry limit. However, exceeding this temperature threshold would result in corrosion rates surpassing the recommended limits (Table 5).

Another potential application for S32101 stainless steel is in the bleaching plant, where the maximum corrosion rate at 50 °C was only 0.002 mm/yr, well below the industry limit. Further reduction in temperature to 20 °C would lower the corrosion rate by potentially a factor of 1200. For S32101 stainless steel in the pulp liquors, the black liquor induces the least corrosive detriments with the corrosion rates (~0.005 mm/year) at all temperatures falling well below the maximum permissible limit, presenting another opportunity for application. Additionally, the current practice in a South African pulp and paper mill involves using fibreglass (amongst other materials: fibre plastic, Grade 2 Titanium, and PTFE-lined carbon steel) across the bleaching section and 316L across the weak black liquor handling units. Given that the overall mechanical properties of fibreglass are inferior to that of S32101 stainless steel, especially in terms of toughness and ductility, replacing fibreglass with S32101 could provide enhanced structural integrity and durability.

On the other hand, S32101 stainless steel is not recommended for storing or transporting black, green, and white liquors due to the high corrosion rates observed at all temperatures, which exceed the acceptable limits in the pulp and paper industry.

## 5. Conclusions

The corrosion performance of S32101 stainless steel, a cost-effective alternative to traditional 304 and 316L stainless steels, was evaluated in various electrolytes representative of the pulp and paper industry. Electrochemical testing techniques and scanning electron microscopy were employed to gain insights into the corrosion behaviour of this alloy in different environments. The objective was to establish guidelines for its suitability in specific plant sections. Based on the experimental results, the following conclusions were drawn:

Corrosion Behaviour in Sulphuric and Chloride-Containing Solutions:

- S32101 primarily undergoes uniform corrosion in sulphuric acid but experiences pitting corrosion in chloride-containing solutions, whether acidic or salt-based.
- Corrosion rates are significantly high in these environments and exceed the recommended limits for structural alloys, particularly at elevated temperatures, which further exacerbate degradation.

Performance in Black, Green, and White Liquors:

- S32101 exhibits superior corrosion resistance in black liquor, while green and white liquors lead to the highest corrosion rates.
- Corrosion rates are roughly constant in black liquor but increase with temperature in green and white liquors; however, they remain significantly lower than in sulphuric acid and chloride solutions.
- The inferior corrosion performance in white liquor is attributed to NaOH, the primary constituent, which can dissolve the passive chromium oxide film when its concentration exceeds 1 mol/L.
- S32101 is recommended for use in black liquor environments, where lower NaOH concentrations and the presence of organic substances help inhibit corrosion. It is proposed as a substitute for the currently applied 316L stainless steel.

Suitability for Bleaching Solutions:

- S32101 demonstrates excellent corrosion resistance in pulp and paper bleaching solutions, particularly those containing chlorine dioxide, a strong oxidising agent.
- Compared to fibreglass, currently used in the bleaching section or other expensive alternatives like Grade 2 Ti and PTFE lined carbon steel, S32101 offers enhanced mechanical strength and extended service life, making it a more durable alternative for this application.

S32101 stainless steel presents promising corrosion resistance in selected environments within the pulp and paper industry. While its performance is limited in highly aggressive acidic and chloride-rich conditions, it is well-suited for applications in black liquor and bleaching solutions, offering both cost and durability advantages. Future work should assess the corrosion performance of their weld regions and heat-affected zones to guarantee their safe use in the pulp and paper industry.

## Figures and Tables

**Table 1:** Composition of S32101 stainless steel.

Element	C	Cr	Ni	Mo	Mn	Si	Co	Cu	N	P	S	Fe
Amount (wt%)	0.031	21.4	1.56	0.19	4.71	0.68	0.058	0.33	0.23	0.03	0.02	Balance

**Table 2:** Typical composition of pulping liquors [13].

Compound	Concentration (g/L)		
	White	Green	Black
NaOH	90	19.1	1.1
Na_2_S	39	42.7	6.2
Na_2_CO_3_	26.2	134.9	1.23
Na_2_SO_4_	8	8.7	2.13
Na_2_S_2_O_3_	4	7.1	0.10
Na_2_SO_3_	0.9	1.4	-
pH	13.5~14	10~14	10~13.5

**Table 3:** Corrosion parameters of S32101 in 1 M H_2_SO_4._

Electrochemical Parameter	Temperature			
	20 °C	30 °C	40 °C	50 °C
Ecorr (V)	−0.47 ± 0.13	−0.42 ± 0.004	−0.43 ± 0.12	−0.43 ± 0.017
icorr (µA/cm^2^)	661.4 ± 27.0	1042 ± 627	1277 ± 108	1378 ± 266
Eprim.pass (mV)	−436	−417	−295	−384
icrit (mA/cm^2^)	1.94	5.34	25.4	10
Epass (mV)	291	302	450	213
ipass (µA/cm^2^)	0.009	0.010	0.134	0.014
Ebd(pit) (mV)	874	981	896	828
Ba (V/dec)	0.057 ± 0.017	0.125 ± 0.0003	0.261 ± 0.089	0.019 ± 0.026
Bc (V/dec)	0.093 ± 0.008	0.054 ± 0.067	0.253 ± 0.062	0.972 ± 0.167
CR (mm/year)	7.0 ± 0.29	11.0 ± 0.66	13.5 ± 1.1	14.4 ± 0.2

**Table 4:** Corrosion parameters of S32101 in 0.1 M HCl.

Electrochemical Parameter	Temperature			
	20 °C	30 °C	40 °C	50 °C
Ecorr (V)	−0.42 ± 0.02	−0.43 ± 0.01	−0.45 ± 0.01	−0.41 ± 0.02
icorr (µA/cm^2^)	15.3 ± 8.2	17.3 ± 0.9	20.4 ± 4.6	367.9 ± 62.6
icrit (µA/cm^2^)	0.004	0.021	0.019	0.035
ipass (µA/cm^2^)	0.006	0.034	0.018	0.035
Ebd(pit) (mV)	479	180	9	92
Ba (V/dec)	0.342 ± 0.087	0.276 ± 0.157	0.266 ± 0.030	0.117 ± 0.002
Bc (V/dec)	0.116 ± 0.035	0.100 ± 0.001	0.148 ± 0.039	0.094 ± 0.007
CR (mm/year)	0.16 ± 0.09	0.18 ± 0.009	0.22 ± 0.05	3.9 ± 0.66

**Table 5:** Corrosion parameters of S32101 in 3.5% NaCl.

Electrochemical Parameter	Temperature			
	20 °C	30 °C	40 °C	50 °C
Ecorr (V)	−0.23 ± 0.001	−0.51 ± 0.11	−0.38 ± 0.04	−0.53 ± 0.13
icorr (µA/cm^2^)	0.3 ± 0.09	1.0 ± 0.4	11.2 ± 8.9	16.1 ± 6.8
icrit (µA/cm^2^)	0.89	36.4	166.7	45.9
ipass (µA/cm^2^)	1.0	28.9	119.7	31.7
Ebd(pit) (mV)	687.3	46.7	−340	102
Ba (V/dec)	0.531 ± 0.196	0.194 ± 0.074	0.660 ± 0.220	0.363 ± 0.124
Bc (V/dec)	0.068 ± 0.002	0.062 ± 0.019	0.072 ± 0.011	0.060 ± 0.001
CR (mm/year)	0.003 ± 0.001	0.01 ± 0.01	0.12 ± 0.13	0.17 ± 0.09

**Table 6:** Corrosion parameters of S32101 in 3.5% NaCl + 0.1 M HCl.

Electrochemical Parameter	Temperature			
	20 °C	30 °C	40 °C	50 °C
Ecorr (V)	−0.25 ± 0.07	−0.48 ± 0.02	−0.40 ± 0.03	−0.45 ± 0.01
icorr (µA/cm^2^)	1.6 ± 0.2	23.5 ± 6.1	337 ± 108	379 ± 49.2
icrit (µA/cm^2^)	1.67	35.4	-	62.8
ipass (µA/cm^2^)	1.27	35.7	-	27.1
Ebd(pit) (mV)	445	7.78	−18.9	−154
Ba (V/dec)	0.172 ± 0.054	0.013 ± 0.012	0.079 ± 0.013	0.421 ± 0.167
Bc (V/dec)	0.129 ± 0.021	0.014 ± 0.015	0.148 ± 0.027	0.052 ± 0.005
CR (mm/year)	0.02 ± 0.01	0.25 ± 0.06	3.6 ± 0.53	4 ± 1.15

**Table 7:** Corrosion parameters of S32101 in weak black liquor (WBL).

Electrochemical Parameter	Temperature			
	20 °C	30 °C	40 °C	50 °C
Ecorr (V)	−0.70 ± 0.02	−0.75 ± 0.05	−0.65 ± 0.11	−0.72 ± 0.08
icorr (µA/cm^2^)	0.12 ± 0.83	0.55 ± 0.14	0.41 ± 0.26	0.48 ± 0.24
icrit (µA/cm^2^)	3.30	6.62	3.30	3.30
Epass (mV)	−451	−451	−451	−541
ipass (µA/cm^2^)	4.05	5.85	4.05	4.05
Etrans (mV)	637	621	597	559
Ba (V/dec)	0.200 ± 0.002	0.057 ± 0.070	0.061 ± 0.061	0.182 ± 0.075
Bc (V/dec)	0.081 ± 0.020	0.160 ± 0.013	0.115 ± 0.003	0.073 ± 0.008
CR (mm/year)	0.001 ± 0.001	0.006 ± 0.002	0.005 ± 0.03	0.006 ± 0.03

**Table 8:** Corrosion parameters of S32101 in green liquor (GL).

Electrochemical Parameter	Temperature			
	20 °C	30 °C	40 °C	50 °C
Ecorr (V)	−1.11 ± 0.003	−1.14 ± 0.007	−1.15 ± 0.009	−1.16 ± 0.040
icorr (µA/cm^2^)	19.7 ± 4.4	29.0 ± 8.2	38.1 ± 35.9	155 ± 27.5
icrit (µA/cm^2^)	57.16	90.22	69.76	91.22
Epass (mV)	−912	−1032	−1048	−1062
ipass (µA/cm^2^)	27.13	71.58	44.62	27.45
Etrans (mV)	−352	−383	−394	−373
Ba (V/dec)	0.204 ± 0.050	0.106 ± 0.016	0.127 ± 0.016	0.133 ± 0.119
Bc (V/dec)	0.211 ± 0.037	0.127 ± 0.002	0.253 ± 0.057	0.439 ± 0.210
CR (mm/year)	0.21 ± 0.05	0.31 ± 0.09	0.40 ± 0.40	1.6 ± 0.29

**Table 9:** Corrosion parameters of S32101 in white liquor (WL).

Electrochemical Parameter	Temperature			
	20 °C	30 °C	40 °C	50 °C
Ecorr (V)	−0.75 ± 0.03	−0.81 ± 0.15	−0.80 ± 0.02	−0.80 ± 0.01
icorr (µA/cm^2^)	122 ± 36.7	562 ± 244	612 ± 232	1695 ± 617
icrit (µA/cm^2^)	107.1	300.3	501.9	566.3
Epass (mV)	−652	−654	−661	−664
ipass (µA/cm^2^)	82.9	158	198.3	302.8
Etrans (mV)	387	397	403	446
Ba (V/dec)	0.622 ± 0.174	0.121 ± 0.019	0.109 ± 0.010	0.179 ± 0.018
Bc (V/dec)	0.076 ± 0.005	0.181 ± 0.019	0.118 ± 0.050	0.287 ± 0.078
CR (mm/year)	1.3 ± 0.39	5.9 ± 2.5	6.5 ± 2.5	12.4 ± 4.5

**Table 10:** Corrosion parameters of S32101 in chlorine dioxide.

Electrochemical Parameter	Temperature			
	20 °C	30 °C	40 °C	50 °C
Ecorr (V)	0.02 ± 0.04	0.01 ± 0.02	0.01 ± 0.02	−0.04 ± 0.06
icorr (µA/cm^2^)	0.01 ± 0.07	0.03 ± 0.07	0.07 ± 0.01	0.17 ± 0.38
icrit (µA/cm^2^)	0.344	0.853	3.53	2.95
Epass (mV)	496	198	489	879
ipass (µA/cm^2^)	0.933	0.741	3.37	25.9
Etrans (mV)	1425	1320	1405	1279
Ba (V/dec)	0.022 ± 0.257	0.015 ± 0.068	0.015 ± 0.022	0.101 ± 0.039
Bc (V/dec)	0.013 ± 0.025	0.028 ± 0.124	0.028 ± 0.007	0.103 ± 0.005
CR (mm/year)	0.0001 ± 0.0005	0.0003 ± 0.0003	0.0007 ± 0.0001	0.0018 ± 0.004

## Data Availability

The data that support the findings of this study are available from the corresponding author upon reasonable request.
